# Towards Interpretable Deep Learning: A Feature Selection Framework for Prognostics and Health Management Using Deep Neural Networks

**DOI:** 10.3390/s21175888

**Published:** 2021-09-01

**Authors:** Joaquín Figueroa Barraza, Enrique López Droguett, Marcelo Ramos Martins

**Affiliations:** 1 LabRisco—Analysis, Evaluation and Risk Management Laboratory, Department of Naval Architecture and Ocean Engineering, University of São Paulo, São Paulo 05508-030, Brazil; mrmartin@usp.br; 2 Department of Civil and Environmental Engineering & The Garrick Institute for the Risk Sciences, University of California, Los Angeles, CA 90095, USA; eald@ucla.edu

**Keywords:** feature selection, deep learning, deep neural networks, prognostics and health management, interpretable AI

## Abstract

In the last five years, the inclusion of Deep Learning algorithms in prognostics and health management (PHM) has led to a performance increase in diagnostics, prognostics, and anomaly detection. However, the lack of interpretability of these models results in resistance towards their deployment. Deep Learning-based models fall within the accuracy/interpretability tradeoff, which means that their complexity leads to high performance levels but lacks interpretability. This work aims at addressing this tradeoff by proposing a technique for feature selection embedded in deep neural networks that uses a feature selection (FS) layer trained with the rest of the network to evaluate the input features’ importance. The importance values are used to determine which will be considered for deployment of a PHM model. For comparison with other techniques, this paper introduces a new metric called ranking quality score (RQS), that measures how performance evolves while following the corresponding ranking. The proposed framework is exemplified with three case studies involving health state diagnostics and prognostics and remaining useful life prediction. Results show that the proposed technique achieves higher RQS than the compared techniques, while maintaining the same performance level when compared to the same model but without an FS layer.

## 1. Introduction

During the last decade, Deep Learning (DL) algorithms have gained great popularity in various areas. Though their theoretical foundations were developed mostly between the 1940s and the 1970s [1,2,3,4,5,6], technological limitations presented in [1] hindered their progress and research went into a hiatus. Nowadays, areas such as natural language processing [7], healthcare [8,9,10], computer vision [11], autonomous driving [12], among others, have embraced machine learning (ML) DL-based models in order to reach better performance. For example, in healthcare, DL models are used for diagnosing and predicting diseases [8,9,10,13,14,15,16,17]. In prognostics and health management (PHM), much like in healthcare, ML and DL models have been used for diagnostics [18,19,20,21,22,23,24,25], prognostics [26,27,28,29,30,31,32] and anomaly detection [33,34] in machinery, showing promising results. In [25], the author proposes a technique called BCAoMID-F (Binarized Common Areas of Maximum Image Differences—Fusion) for feature extraction using thermal images. This technique is applied for fault diagnosis in angle grinders using support vector machines (SVM) and nearest neighbors (NN). In [21], Verstraete et al. used convolutional neural networks (CNN) for fault diagnosis in rolling element bearings, feeding the network with image representations of the raw vibration signal, thus, leaving the feature extraction process to the network. In [24], authors used a recently developed DL architecture called capsule networks to identify and quantify structural damage using transmissibility measures converted to images. They achieved better performance than neural networks and CNNs, also achieving better generalization. In [31], Zhang et al. used long short-term memory networks (LSTM) to calculate the remaining useful life (RUL) of turbofan engines, taking advantage of the benefits of LSTM when working with time series.

Despite DL showing promising results, there is hesitancy in adopting these models due to their black-box nature. Their high level of complexity impedes a proper interpretation of the results, which is an important issue in many areas such as safety critical applications. In Europe, the General Data Protection Regulation (GDPR), which took effect as of 2018, includes a right to explanation regarding algorithmic decision-making. This means that, in a situation where a decision that significantly affects someone is made based on an algorithm, that person has the right to know how and why that decision has been made. A clear example of this is loan application. Organizations are struggling to comply with this regulation [35] because there are only few algorithms whose results can be interpreted. Unfortunately, these fewer complex algorithms do not show results as promising as DL-based models. Furthermore, there is no consensus on what an appropriate explanation is. To address this last issue, researchers have proposed different taxonomies to organize concepts and techniques [36,37,38]. Most recently, Fan et. al. [37] proposed a taxonomy in which techniques are primarily divided according to when are they applied, with respect to the training process. They identified techniques that are applied after training using external tools (referred to as post hoc analysis), and those that modify the inner dynamics of the model for it to be more easily understood (referred to as ad hoc modeling). This taxonomy serves as a first step towards a concrete definition of interpretability and how to measure it. Among post hoc techniques, they identified approaches such as feature analysis, model inspection, saliency maps, proxy models, mathematical and/or physical analysis, and explanations by case and by text. In turn, ad hoc techniques are classified into interpretable representation through regularizations and model renovations through interpretable parameters. Comparing post hoc with ad hoc techniques, the first kind relies on approaches that generate surrogate interpretable models that approximate the original model’s behavior. The drawback is that through approximation there is no access to the inner dynamics of the model. Thus, the surrogate model may generate explanations that do not correspond with the inner dynamics of the original model. Furthermore, results generated by post hoc techniques have an associated error, as they only approximate the behavior of the original model. In contrast, ad hoc techniques access the inner dynamics of the model. However, there is a limited number of algorithms that allow an analysis of their inner dynamics (commonly referred to as opening the black-box), and are usually less complex algorithms, such as linear regression, rule-based models and logistic regression. As models get more complex, they yield better results but have a decreased capacity for interpretability. This is known as the accuracy/interpretability tradeoff. It has been analyzed in the literature [38,39,40,41] and is the main reason why post hoc techniques are more popular than ad hoc techniques, even though only the latter open the black box. Neural networks are complex algorithms that reach high levels of performance but fall within the accuracy/interpretability tradeoff and, thus, lack of interpretability. This issue with performance and interpretability must be tackled by creating techniques that increase the interpretability of neural networks without dropping performance.

In the context of PHM, interpretability is necessary to build trustworthy models. For a company to rely on a DL model to diagnose and/or predict a physical asset’s health states, it is natural for people to demand not only a satisfactory level of performance but also an understanding of how the model works. In [42], Rezaeianjouybari and Shang conduct an extensive review of DL in PHM, stating there is a challenge for researchers in the area of developing techniques for model interpretation to overcome the unwillingness of some companies to adopt DL.

According to the authors in [38], model interpretability frameworks can be divided into local and global interpretability. While local interpretability aims to explain single predictions, global interpretability aims to give an interpretation of how the model features interact to make predictions. Two of the most accepted frameworks for interpretability that can be used in DL models are the Local Interpretable Model-agnostic Explanation (LIME) [43] and Shapley Additive Explanations (SHAP) [41] algorithms. LIME attempts to explain a single prediction by approximating the model locally using perturbations of the corresponding datapoint. SHAP adapts the Shapley values developed for game theory to determine what is the contribution of each feature in a single prediction. Both techniques are model agnostic, meaning they can be used for any model, including neural networks. However, they are post hoc techniques, meaning they require a trained model for analysis, and rely on input perturbations. This makes them prone to output misleading results in certain situations, such as adversarial attacks, as discussed in [44]. Since they are algorithms based on perturbations, results can be negatively affected if these perturbations come from a source different than the phenomenon trying to be analyzed (for example, degradation).

Within global interpretability, feature selection is a relevant aspect, as it indicates how much participation each variable has within the model. When done before training, feature selection is used to select the most relevant features to feed the model. It also helps in detecting irrelevant features, which reduces overfitting and may lead to an improvement in performance. Furthermore, a model becomes easier to comprehend when it has less variables. There are numerous methods for feature selection, which, according to the literature [45] can be divided into three categories: wrapper, filter, and embedded methods. Wrapper methods are those where the model is trained with different combinations of input features to determine which gives the best results. These methods clearly lack scalability due to time consumption [46]. Filter methods use statistical metrics before training for feature selection. Examples of these metrics are the Pearson correlation coefficient, χ^2^ test, and mutual information. These kinds of models do not relate to the model or its predictions after training. In addition, they only analyze the dependence of each feature with the output individually, not considering the interactions among features [47]. An example is shown in [9], where the authors use a combination of mutual information analysis and wrapper methods to delete the least relevant features for heart disease prediction using an ensemble deep learning model. In [10], the authors use the χ^2^ test to select the ten most relevant features for cervical cancer prediction using isolation forest. Embedded methods refer to algorithms with built-in techniques for selecting features. They correspond to ad hoc modelling. These include Random Forests (RF), Lasso Regression and Ridge Regression, among others [48,49,50]. The drawback of these kinds of techniques is that they cannot be detached from the algorithm. In addition, neural networks do not have an intrinsic feature selection technique. Thus, an embedded technique cannot be used in a neural network without training more than a single model.

Despite the fact of neural networks not having an embedded technique for feature selection, wrapper and filter methods can still be used. However, the objective of DL models is to use the least data preprocessing possible. Neural networks have an important number of hyperparameters to be tuned which turn the search for an appropriate model into a slow and intricate process. A data preprocessing stage makes this process even slower. In addition, the use of wrapper or filter methods on DL models would amplify their aforementioned drawbacks. To address these issues, researchers have studied different ways to adapt DL models for feature selection [51,52,53,54,55,56,57,58,59]. In [51], Chang et al. use variational dropout in the input layer as a means to determine feature importance. In their approach, the individual dropout rate for each feature indicates how much the model is allowed to remove that feature. Thus, features with low dropout rate are more relevant than those with a high dropout rate. These values are used to build a ranking of features. They test their approach in two simulated datasets and six real-world datasets from diverse areas (none of them related to PHM) and achieve results similar to those using Random Forest for ranking features. In [58], the authors propose an approach based on attention mechanisms. It consists of a feature weights generation module (also called “attention module”) made of parallel hidden layers incorporated to the neural network next to the input layer. Each part of the module outputs a value between 0 and 1, which is then multiplied by its corresponding feature to enter the rest of the network, which they call the “learning module”. They test their approach using the MNIST dataset [60], the noisy-MNIST dataset with its three variants [61] and two other small datasets used for feature selection problems [62]. Results show their framework achieves better results when compared with other filter and embedded methods.

In this paper, we propose a framework for feature selection embedded in deep neural networks (DNN) for PHM in which a feature selection (FS) layer is added next to the input layer to be trained jointly with the rest of the fault diagnosis and prognosis network. The main contributions of this work are the following:

- An in-model technique, referred to as feature selection layer (FS) technique, for DNN feature selection, which helps interpreting the model without performance decrease. It uses the DNN’s inner dynamics to determine each feature’s importance without the need of an external technique. It is an ad hoc technique that addresses the accuracy/interpretability tradeoff.
- A framework for ranking quality evaluation based on a new metric, which is defined as ranking quality score (RQS) that performs a quantitative evaluation of the feature ranking obtained through the FS layer technique. It is also used to allow a fair comparison of the proposed framework with other techniques.
- Application of the FS layer technique for fault diagnosis and prognosis (classification) and RUL (regression) prediction tasks for PHM in four different case studies.
- Comparison of the proposed technique with other filter and embedded methods.

The remainder of the paper is organized as follows. In Section 2, a brief theoretical description of deep neural networks is presented. In Section 3, the case studies used in this work are presented and described. The proposed framework for feature selection to improve the DNN model interpretability and ranking quality evaluation are presented in Section 4. Section 5 presents and discusses the obtained results. Concluding remarks are presented in Section 6.

## 2. Deep Neural Networks

**Figure 1:**
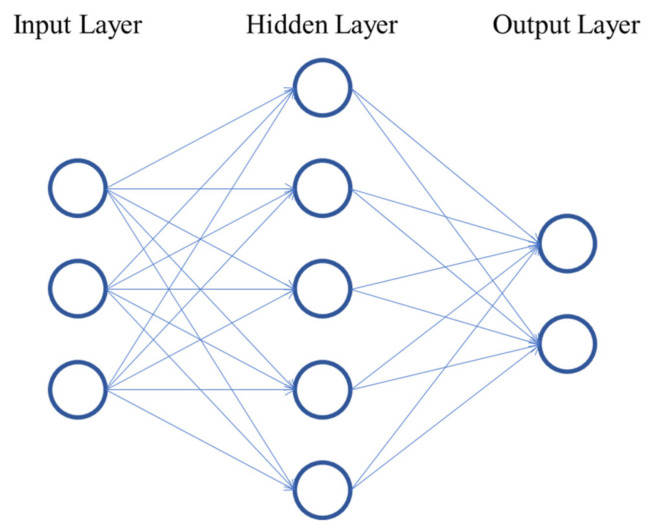
Neural network representation.

Within Machine Learning algorithms, Artificial Neural Networks (ANN) have stood out in recent years due to their state-of-the-art results in many domains and their processing capabilities. An ANN is a data-driven model used to encode a phenomenon. The structure of an ANN is presented in Figure 1. It consists of neurons organized into layers and is inspired in the way neurons interact in the brain to process information from the environment. In the figure, each node in the input layer represents one feature. They are linearly combined using trainable weights (represented by the connections between nodes) to output the values of the neurons into the next layer. To add nonlinearity to the model, these values are passed through an activation function. Finally, a trainable bias term is added. Thus, the values in each layer (except the input layer) are defined as:

(1) $$a_{i}^{j}=f\left(\sum_{k}w_{k,i}^{j}a_{k}^{j-1}+b^{j-1}\right),$$

where $a_{i}^{j}$ is the value (also called activation) of the i-th neuron in the j-th layer, $w_{k,i}^{j}$ is the trainable weight connecting the k-th neuron in the previous layer with the i-th neuron in the current layer, $a_{k}^{j-1}$ is the activation of the k-th neuron in the previous layer, $b^{j-1}$ is the bias term of the previous layer and f corresponds to the activation function of the current layer. ANNs can be divided by the type of task: classification or regression. In classification tasks, the output layer has one neuron per class. The one with the highest activation value indicates the class to which the model associates the corresponding input. In regression tasks, the output layer has one neuron, which corresponds to the continuous value the model calculates. An ANN is optimized through backpropagation [63], in which the weights and biases in the network are modified to minimize a loss function. Hyperparameters such as number of hidden layers, number of neurons per layer, activation functions of each layer, loss function and optimization algorithm, among others, must by fine-tuned to achieve higher performance.

Though ANNs include only one hidden layer, the addition of more hidden layers results in models able to find more complex dependencies in the data and thus achieve higher performance. These kinds of networks with multiple hidden layers are referred to as Deep Neural Networks (DNN) and define the subset of Machine Learning algorithms called Deep Learning.

Due to the stacking of several hidden layers, DNNs are able to model complex and rare dependencies in the training data. However, sometimes this ability can lead to overfitting, as the model finds relations within the data that cannot be extrapolated to new observations. There are several ways to address this issue, including parameter penalty terms, dataset augmentation, multitask learning, early stopping and dropout [64], among others. Regarding techniques based on penalty terms, two of the most used ones are the $L^{1}$ [65] and $L^{2}$ [66,67] regularization. In DL, the $L^{1}$ regularization refers to the addition of a penalizing term to the loss function as

(2) $$L^{1}\left(k\right)=\lambda \cdot \underset{i}{\sum }\left|w_{i}^{k}\right|$$

where λ determines the strength of the regularization and $w_{i}^{k}$ is the i-th weight in the k-th layer. Thus, when an $L^{1}$ regularization is applied to a certain layer of the network, the model, along with the primary task, tries to minimize the $L^{1}$ norm of the layer’s weights. On the other hand, the $L^{2}$ regularization applies the same principle but with the $L^{2}$ norm:

(3) $$L^{2}\left(k\right)=\lambda \cdot \sqrt{\underset{i}{\sum }{\left|w_{i}^{k}\right|}^{2}}$$

**Figure 2:**
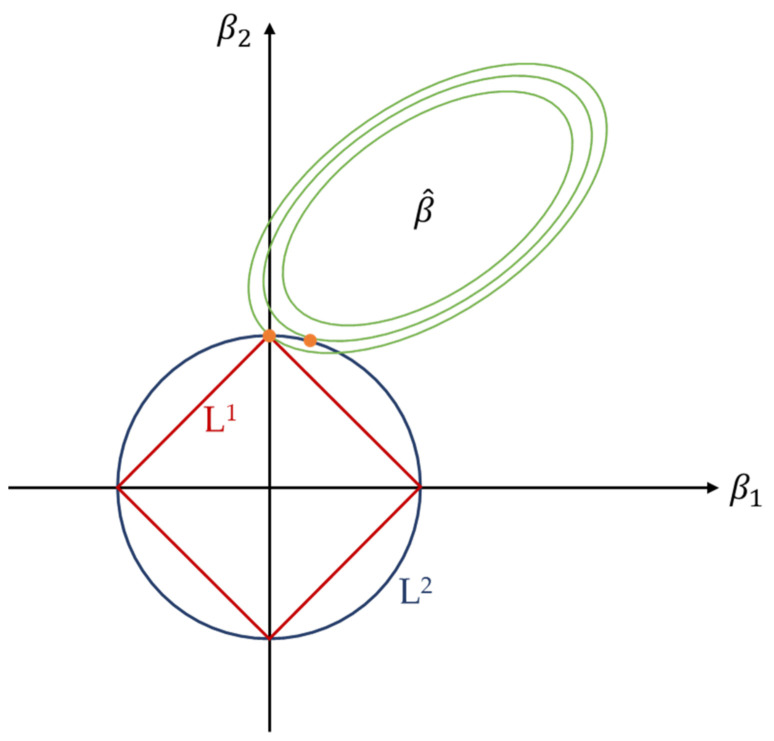
Least squares solution contour for a linear model with $L^{1}$ (red curve) and $L^{2}$ (blue curve) regularizations. Based on [71].

Though these two techniques are similar, their effect on the model and the learning process is different. According to [68], due to their derivatives, both encourage small weight values, but weights regularized by $L^{2}$ seldom reach the value zero, whereas $L^{1}$ induces solutions where a few number of weights have values greater than zero. This is explained in [65]. According to the author, this is due to the shape of the constrain region of the regularizers. This is shown in Figure 2. This sparsity property of the $L^{1}$ regularization has been used for feature selection tasks [69]. For example, in [70], Ng claims that in the context of logistic regression, a model is less affected by irrelevant features when it is regularized using $L^{1}$ regularization than when using $L^{2}$ regularization. When $L^{1}$ is used, the number of samples needed for an acceptable level of performance grows with the number of irrelevant features at a logarithmic rate, whereas with $L^{2}$ regularization, this rate is linear.

## 3. Case Studies

This section presents the case studies used to evaluate the performance of the proposed framework for fault diagnosis and prognosis and RUL prediction.

### 3.1. Case Western Reserve University Bearing Data Center (CWR)

**Figure 3:**
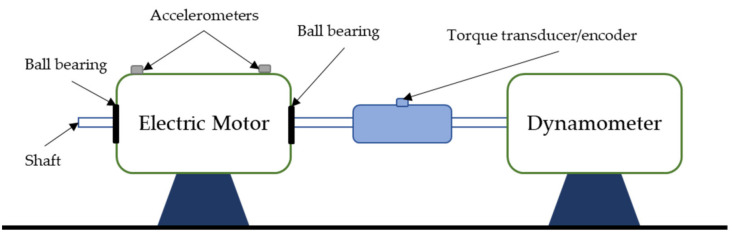
CWR experiment setup. Based on [72].

The dataset was provided by the Case Western Reserve University Bearing Data Center, and consists of vibrational data collected with accelerometers on an electric motor’s drive-end ball bearing [72]. The experiment setup is presented in Figure 3. The description of the experiment is detailed in Table 1. Accelerometers are placed in the bearing housing. Artificial faults are generated using electro-discharge machining and located in the bearing balls, outer ring and inner ring. Twelve health conditions are measured according to the size and location of the generated faults. These determine the bearings’ health classes. Similarly to the work presented in [19], 100 features are manually extracted from the data, which are used as inputs for the model. These are presented in Table 2. The dataset has 8887 samples with 100 features and 12 classes. Eight-thousand and four samples are used for training and validation and 883 are used for testing. This dataset is used within the paper to train a model able to diagnose the bearing’s health state given a combination of input features. Therefore, the definition of the train and test sets is done randomly, without the generation of time windows. The classes’ descriptions are detailed in Table 3.

To diagnose the bearing’s health state, 100 extracted features are used. However, there are features that give more information about the health state than others. Furthermore, a situation may be presented in which the information of some features is obtained through other features, making them irrelevant to the network. Thus, it is necessary to determine the importance of each input feature fed to the network.

Regarding other works, some authors have done feature selection for this dataset [73,74,75,76]. In particular, the authors in [73] propose the use of a modified CNN in which the first layer is built using a second-generation wavelet convolution over the raw signal. Authors in [76] create a framework based in correlation analysis, principal component decomposition (PCA) and weighted feature fusion in order to train a model over extracted features from the raw signal. They successfully identify useful features to train an SVM. However, their approach is not ad hoc; therefore, it does not access the inner dynamics of the model.

### 3.2. Commercial Modular Aero-Propulsion System Simulation (C-MAPSS)

The second case study used in this work corresponds to the NASA Commercial Modular Aero-Propulsion System Simulation (C-MAPSS) datasets [77], widely used as benchmark for RUL prediction methods [29,30,31,78,79,80,81]. They are four datasets containing simulated data of turbofans degradation time series. The datasets descriptions are shown in Table 4. As noted in the table, data are already divided into train and test trajectories. In the case of the training set, each trajectory ends in failure. In the test set, each trajectory ends in a point before reaching the failure threshold. In both cases, trajectories differ from each other in their initial wear, so they can characterize different forms of fault evolutions. In addition, operating conditions in the datasets alternate between 1 and 6 and fault modes alternate between 1 and 2, making each dataset different from the others. Each dataset is comprised of 21 sensor measurements, which are used as inputs to train models for RUL prediction. Each sensor measurement is intentionally polluted with noise in order to emulate a real-case scenario. To generate the labels, the same procedure shown in [29] is used. Thus, degradation is represented as a piecewise linear function of the time cycles, with a maximum RUL number of 125. A diagram of the engine used for creating the datasets and the sensor points is shown in [82]. A description of the features used for RUL estimation is given in Table 5.

Out of the four datasets, this work shows results on datasets FD001 and FD004, since this work’s objective is to show the framework’s performance in different scenarios, and these two datasets do not share any of the characteristics shown in Table 4. The sizes of the two datasets are shown in Table 6.

As mentioned before, the C-MAPSS datasets are used for RUL prediction. Typically, best results are obtained when using CNNs and/or LSTMs [31,79,80,81]. Here, the data are organized into time windows for the model to learn to associate a certain evolution of feature values to a RUL value. In the case of DNNs, inputs are two-dimensional, thus, there is no construction of time windows. This naturally hinders the results obtained when using DNNs. However, the objective of this work was not to improve the state-of-the-art results on RUL prediction but to present a framework for interpretability of DNNs in the context of PHM that does not fall into the accuracy/interpretability tradeoff. Hence, the use of DNNs is not an issue within this work.

Among the input features, there are some more informative about the degradation of the turbofan than others. This means that some features (or combination of features) are more sensitive to a change in the health state than others. More specifically, there may be features that are related to a certain failure mode that do not relate to another failure mode. Therefore, it is relevant to determine the importance of each input feature on both datasets.

Regarding other works, the authors in [83] do an extensive analysis of feature selection approaches applied to these datasets, including filter and wrapper methods. They argue that these methods reduce model complexity; however, they do not guarantee an improvement in performance. This relates to the fact that filter methods are detached from the model itself, and may generate results that do not apply to a specific model. For wrapper methods, several different models are trained according to different sets of features. This generates misleading interpretations of the results, as different models may use features in different ways.

### 3.3. Offshore Natural Gas Treatment Plant

**Figure 4:**
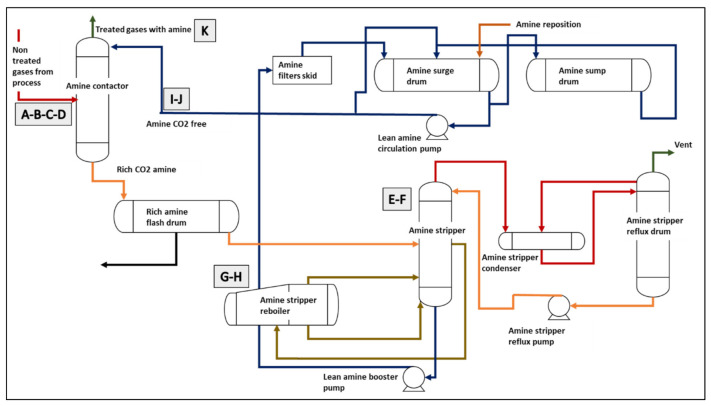
CO_2_ removal process illustration. Source: [32].

In this case study, data comes from an offshore natural gas treatment plant used to remove CO_2_ from the gas using amines. This process, also known as “gas sweetening”, is done because CO_2_ is corrosive and reduces the natural gas energetic value. It is also used to remove H_2_S, which is toxic and corrosive. Figure 4 shows a schematic representation of the plant. The CO_2_ removal is done in the amine contactor, where nontreated gas and the amines flow in countercurrent, so that the amines capture CO_2_. The rest of the system has the function of removing the CO_2_ from the amines so it may be used again. The rich amine (amine containing CO_2_) enters a flash drum in which small amounts of hydrocarbons typically present in the solution are separated. Then, the amine enters the amine stripper. There, the rich amine contacts vaporized water to be heated and stripped out from CO_2_. The reboiler is used to help this process by boiling the rich amine from the stripper and also providing the stripper with vaporized water. The lean amine from the bottom of the stripper then goes to the amine filters skid, surge drum and sump drum for recycling and recirculation in the contactor. The overhead vapor (water vapor and gas containing CO_2_) from the top of the stripper enters a condenser. The condensed vapor then enters a reflux drum in order to separate water (which is reused in the stripper) from acid gas, which goes to disposal.

To monitor the plant’s performance, 10 variables are measured constantly. Table 7 shows a description of the features, which are also identified in Figure 4. This helps to establish relations between some features. For example, feature D should have a similar value to the difference between the values of features B and I. The objective is to reduce the CO_2_ levels in the treated gas to <2500 ppm, which is the acceptable level. The dataset contains 5915 points sampled every 20 min, divided into four time periods. It is used to train models for quantifying the CO_2_ levels after gas treatment. The input data corresponds to features A to J from Table 7 while feature K is used to calculate the model’s output error. As in the CWR case, the data is split into train and test sets randomly, with 4732 samples used for training and 1183 used for testing. This can be done because no time windows are generated.

In this case study, input features are related to certain equipment acting on the process. To identify an irrelevant variable could lead to stopping the monitoring of equipment that does not need monitoring. On the other hand, identifying a feature with high relevance could lead to identifying equipment that has more impact on the amount of CO_2_ in the treated gas. This could help to improve process reliability.

Regarding other works, authors in [32] use an LSTM-based autoencoder to predict whether the amount of CO_2_ will be above or below 2500 ppm after 20 min. They also evaluate how the different performance metrics vary with the prediction time. The feature selection process is done separately from the training process, according to a wrapper method. They use the following features: nontreated gas flow rate (A), nontreated gas temperature (B), amine pressure at the reboiler (H), amine temperature entering the contactor (J) and past measurements of the concentration of CO_2_ in the treated gas (K).

## 4. Proposed Framework

In this section, the proposed framework for feature selection and evaluation is presented. The feature selection (FS) layer and a methodology for evaluating the quality of feature rankings are introduced and discussed. Also, other approaches in the literature are compared with the proposed framework.

### 4.1. Feature Selection Layer

**Figure 5:**
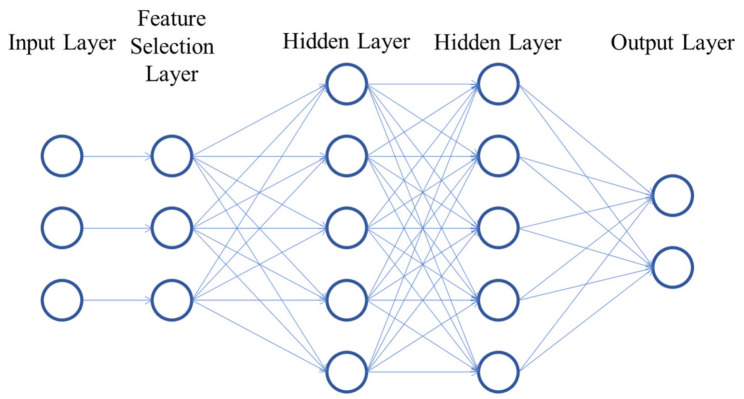
An example of a deep neural network with the proposed FS layer.

The feature importance analysis in DNNs is addressed by adding a feature selection layer (FS) after the input layer, in which each feature is multiplied by a trainable weight with values in the range of [0,1]. No bias is added to the layer. The weights are trained jointly with the rest of the network’s weights and represent each feature’s importance. A representation of this approach, and how it is embedded into the neural network, is shown in Figure 5. Since the objective is to build a technique in which no preprocessing is needed, it is assumed there is no prior information about the features’ importance values. Thus, the weights in the FS layer are initialized as 1/n, where n is the number of features. In addition, a regularization term is added to ensure the interaction between features, which is calculated as:

(4) $$r\left(W^{FID}\right)=\lambda \cdot \left|\overset{n}{\underset{i=1}{\sum }}w_{i}^{FID}-1\right|$$

where $W^{FID}$ represents the weights vector in the FS layer and λ is a value between 0 and 1 that determines the strength of the regularization. The addition of this term to the loss function enforces the weights to sum 1. Thus, it is assured that the importance of each feature is influenced by the interaction with other features, which does not occur in methods like random forest or mutual information. This also avoids situations in which weights have the same values. Furthermore, the use of the $l_{1}$ norm, instead of the $l_{2}$ norm, is based on the effects of the $L^{1}$ and $L^{2}$ regularizations in the model related to feature selection, as detailed in Section 2. This configuration enforces the model to reach sparse solutions in which irrelevant features are tied to a weight value of zero. The use of the $l_{2}$ would result in irrelevant features having values close to zero.

The inclusion of an FS layer to a DNN implies the addition two hyperparameters to the network: the layer’s activation function and the value of the λ parameter presented above. Both must be tuned to reach the highest performance possible, and in unison with the other hyperparameters in the network.

The reason behind the addition of an FS layer is for a DNN to be able to inform how the input features are being used, besides performing its primary task. Since the weights in the FS layer are constrained between 0 and 1, and no bias is added, their values after training indicate how relevant each of the input features are within the model. The layer is regularized by the regularization term shown in Equation (4) to avoid situations in which all the weights have the value 1 or are too close to 0. Thus, the obtained weights can be easily sorted to determine which features are more relevant, without interpretation ambiguity. The λ value is necessary in Equation (4) to avoid a situation in which the regularizing term leads to underfitting.

### 4.2. Ranking Quality Score (RQS) for Ranking Evaluation

**Figure 6:**
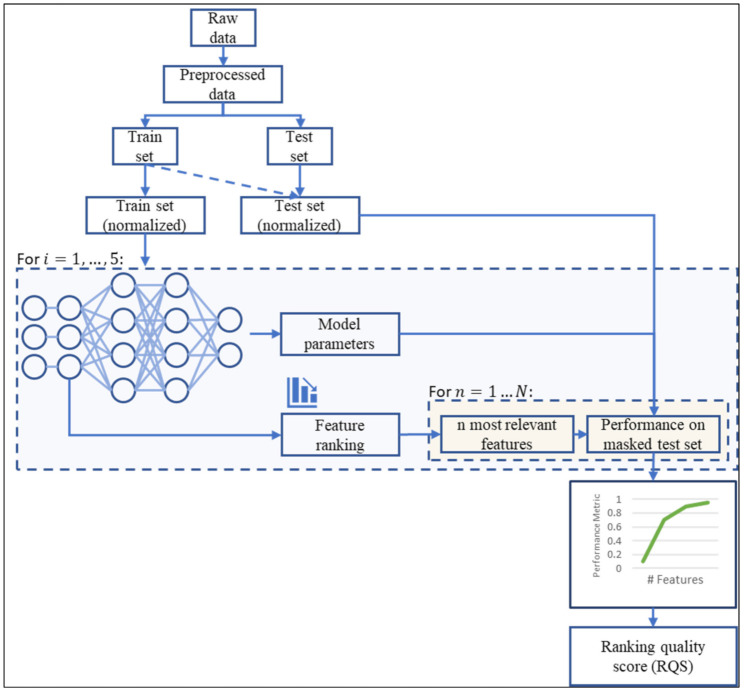
Proposed methodology block diagram for ranking quality evaluation.

The addition of an FS layer to the network enables the creation of a ranking of input features according to their importance values represented by the values of the weight associated to each input. This is useful for visualizing the tradeoff between number of input features and performance, which is key to determining the number of features used in deployment. Here, we propose a framework for a quantitative evaluation of the obtained ranking, which helps on deciding which model is the most suitable, and what features of the input feature space shall be used in the model deployment. This framework is presented in Figure 6. It consists of the following steps:

1. The raw dataset is preprocessed to make it suitable for usage. Since feature selection is done during training, it is not necessary to do any analysis for removing variables, besides discarding categorical variables.
2. The dataset is divided into train and test sets which are normalized. To do this, the scaler chosen to normalize the data is fitted to the train set and used in both the training and testing set. For example, if the data is standardized, the mean and variance used for normalizing both the training and test sets are obtained only with the train set. This is done in order not to have any interference in the training process from the test set.
3. The training set is used for training the network.
4. When the training process is finished, the FS layer’s weights are used to rank the features in decreasing order. The model’s performance is then evaluated in the test set using a subset of the top n features, with $n=\left\{1,2,\ldots ,N\right\}$. At first, the subset only contains the most relevant input feature. After evaluation, the second most relevant feature is added to the subset and performance is evaluated. This process is repeated until all the input features available are added to the subset.

**Figure 7:**
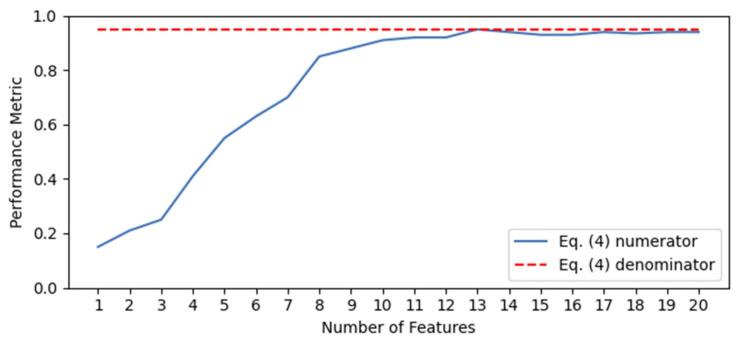
Graphical representation of Equation (5).

**Figure 8:**
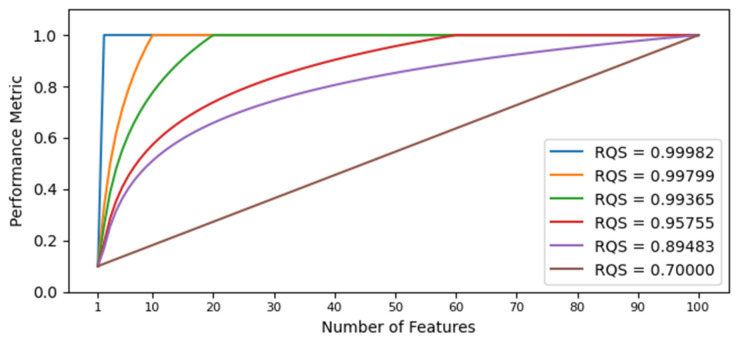
Different ranking situations with their corresponding RQS values.

The performance results obtained from this iterative process are presented in a curve in order to compare with other methods, as in [51,57,58]. However, a visual comparison is not as accurate and reliable as a quantitative comparison. To address this issue, it is proposed the ranking quality score (RQS) is used, which is defined as:

(5) $$RQS\equiv \;\frac{\sum_{n=1}^{N}PM_{n}\cdot n}{\sum_{n=1}^{N}\max\left(PM\right)\cdot n}=\frac{2\cdot \sum_{n=1}^{N}PM_{n}\cdot n}{\max\left(PM\right)\cdot N\left(N+1\right)}$$

where $PM_{n}$ is a performance metric of the choice evaluated using the n most relevant features and $\max\left(PM\right)$ is the maximum value reached by the model. The range of values of the RQS metric is [0,1] (the proof is presented in Appendix A), and it compares the two scenarios exemplified in Figure 7. In the figure, the scenario shown in the blue line represents an example case where the performance metric value increases alongside the number of features. Then, the numerator of Equation (5) is calculated by multiplying each performance metric (calculated through step 4 mentioned above) by the number of features used to reach that performance value and summing up all the results. This value is an indicator of the quality of the ranking obtained through step 4. However, it is highly dependent on the model. Within one model, this value can be used successfully to compare different rankings. Nonetheless, this cannot be done when comparing models. To address this issue, the RQS metric has a normalization term shown in the denominator of Equation (5), represented by a red dotted line in Figure 7. It is the same calculation for the numerator, but assuming an ideal case where the maximum performance value is reached with the most relevant feature and is unaltered by the addition of less relevant features. By using the denominator mentioned above, the RQS metric presents values between 0 and 1. To illustrate its behavior, Figure 8 shows six different curves with their corresponding RQS value.

The RQS metric measures the quality of the ranking obtained through the model using an FS layer. It is a normalized weighted sum of the chosen performance metric. It gives more weight as the number of variables increases. When using one feature, performance typically shows a low value. As features are added, performance increases at a fast rate. After a certain number of features, performance reaches a plateau. The RQS metric gives a high value to a model in which the plateau is reached with few variables, and this plateau is not affected by the addition of more features. On the other hand, it gives a low value to a model in which more features are needed to reach the plateau, or the addition of features leads to an important decrease in performance.

Besides achieving a high RQS, the proposed FS layer technique must, at least, maintain the same level of performance compared to the same network without an FS layer. Otherwise, there would be a tradeoff between performance and interpretability, which is not desirable. To evaluate the influence of the FS layer in the model’s performance, and based in the work presented in [57], relative performance metrics are used, as defined in:

(6) PMn=ρnρ0,  for classification tasks or PMn=R2 ρ0ρn,  for regression tasks with PMn ≠ R2

where $\rho_{n}$ is the performance metric of the model trained with the FS layer and tested with the n more relevant features, and $\rho_{0}$ is the performance metric of the same model but without the FS layer and tested with all the set of features. Since typical regression performance metrics (except the $R^{2}$ coefficient) show the same behavior as the prediction error, the difference in definition is necessary for having the same kind of analysis regardless the task. Performance metrics are used as in Equation (6) to easily illustrate in this work if the inclusion of an FS layer is beneficial or detrimental to the model’s performance. A value >1 indicates the use of an FS leads to an increase in the model’s performance, whereas a value ≤1 indicates the contrary.

The proposed methodology involves the evaluation of models in terms of task performance (through a chosen performance metric) and their feature ranking quality (through the proposed RQS metric) simultaneously. Since the RQS metric quantifies how the model reaches its maximum performance and not the performance itself, this simultaneous analysis is not only possible but necessary. This is more clearly explained by observing that a model may have a good ranking quality (reflected in a high RQS value) but a low task performance. Thus, the selection of an appropriate model (including the subset of input features to be used) is determined by its task performance and its RQS.

### 4.3. Model Selection

The objective of using an FS layer and the RQS metric is to improve the model selection process for DNNs. The RQS metric compares different approaches for feature selection. Thus, a model with a higher RQS value should be preferred over one with a lower RQS value when performance is similar between them. After choosing the appropriate technique, the number of features used in deployment must be determined. To do this, a mathematical-based criterion would be the number of features that yields the maximum performance value. While this is a reasonable criterion, it does not consider the fact that some features may have little impact on performance. In a real-life application, maximum performance may not be the ultimate objective, but rather a performance level above a predefined threshold, subject to limitations such as cost or time, among others. Moreover, models with a high RQS might benefit from other selection criteria besides maximum performance since they achieve high levels of performance with fewer features.

In this work, two criteria for model selection are analyzed. The first one is maximum performance, and the second one is defined as the number of features needed to reach a 95% of the maximum performance value. This is done in order to show the performance and applicability of the techniques in a scenario where maximum performance is required, and another one where a 5% decrease in performance is allowed, emulating a real-case scenario. However, the application of this framework is not restricted to these two criteria. Other criteria can be used for model selection depending on particular objectives and limitations. With this, model selection is done not only evaluating a performance value but also the RQS value simultaneously.

### 4.4. Comparison with Other Techniques

To compare the proposed technique against other approaches in terms of RQS and task performance, the following techniques were tested:

- Mutual Information
- ReliefF [84] and RReliefF [85]
- Random Forest
- “AFS: An Attention-based mechanism for Supervised Feature Selection” [58] (AFS)

In mutual information, feature importance values are determined according to the dependency of the output feature with each input feature, as calculated in Equation (7):

(7) $$MI=\;\underset{x\in X}{\sum }\underset{y\in Y}{\sum }p\left(x,y\right)log\frac{p\left(x,y\right)}{p\left(x\right)p\left(y\right)}$$

where $p\left(x,y\right)$ is the joint probability distribution of features X and Y. Thus, features with which the output is more dependent are more relevant. In ReliefF, features are analyzed in terms of how well they can distinguish classes in instances close to each other. The feature weights are calculated by measuring how the feature value varies with respect to a nearest hit (the nearest instance with the same class) and with respect to nearest misses (nearest instances with different classes). This is repeated for a set of instances. For regression tasks, the ReliefF algorithm is extended to RReliefF [85]. In the case of random forest, feature importance is calculated during training based on how much each feature contributes to the decrease in impurity when splitting data in each tree. For classification tasks, this impurity measure is typically the Gini impurity. For regression tasks, impurity is measured through variance. The splitting process is explained in more detail in [86]. The AFS technique, as described in the Introduction section, presents a detachable module for feature selection based on attention mechanism. An attention net for each feature is trained jointly with the rest of the network to determine whether the feature is relevant or not. The output of each attention net after training is used to rank features.

**Figure 9:**
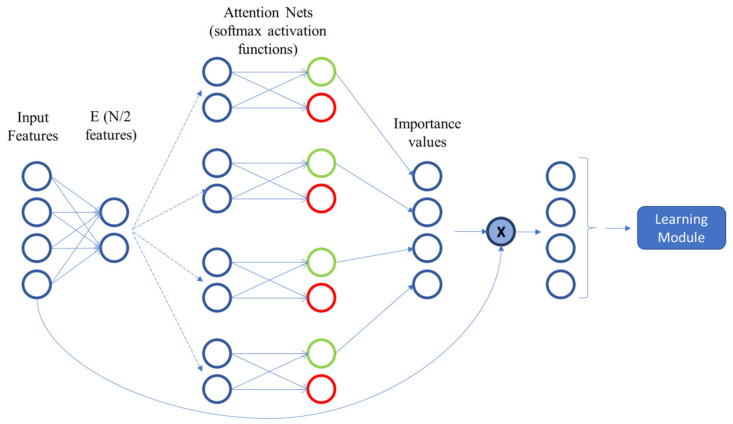
Example representation of the AFS network. Note that the E layer has N/2 neurons. In the AFS2 configuration, the E layer has N features. As described in the original work, the E layer has a tanh activation function. It serves as input to N attention nets, each of them used to determine the importance value of an input feature. Based on [58].

These four techniques cover filter and embedded methods. Wrapper methods are not used since they become unfeasible for some datasets. Mutual information and ReliefF techniques are filter methods, thus they are model agnostic. On the other hand, Random Forest is a popular ML technique, which has an embedded method for feature selection. The AFS method is used for comparison because it is a technique embedded in neural networks, achieving promising results when compared against other filter and embedded techniques, including one used for neural networks referred to in *Roy* et al. [59]. To the best of the authors’ knowledge, the AFS technique is the most promising one amongst those embedded in neural networks. In this paper, we use two different configurations of this model to compare them with the proposed FS layer technique, namely AFS and AFS 2. In the first (AFS), the first hidden layer after the input layer (referred to in the paper as E) has an N/2 neurons, N being the number of input features. A graphical representation of an example (with N=4) is shown in Figure 9. In AFS 2, the same layer has N neurons. Regarding the attention nets, it was decided not to include any hidden layers (referred to in their work as $\left\{h_{1}^{k}\ldots h_{L}^{k}\right\}$) for three reasons: first, the authors in [58] do not specify the number of hidden layers and neurons used in their experiments; second, the implementation available in [87] does not include hidden layers in their attention nets; and last, depending on the number of features, the inclusion of hidden layers would increase the model size and, thus, its computational cost. Therefore, the E layer is connected to N softmax-activated layers with two neurons, which is used to determine the importance of each feature before entering the learning module.

Regarding the two widely used techniques LIME and SHAP discussed in the Introduction section, they are not used for comparison because their mechanism for interpretability uses the model’s predictions. They are post hoc techniques in which explanations depend entirely on the model’s outputs and, therefore, their inputs. In the case of LIME, this is a method for local interpretability, while in the case of the SHAP algorithm, feature importance for the model is obtained through the mean Shapley values for each feature across the dataset. This means that they may eventually vary when evaluating them with other datasets (such as using the test set). In contrast, all of the approaches above estimate feature importance values that do not change when varying the test set.

To evaluate the ranking quality of the aforementioned techniques, the first step is to obtain the features’ importance values using each of them. Then, a neural network with the same configuration as the main model but without the FS layer is trained. After that, similar to the process depicted in Figure 6 the n most relevant features are used to evaluate the network’s performance on the masked test set, with n varying from 1 to the total number of features N. The network’s performance evolution across the number of features is represented in a curve, and RQS is calculated, as discussed in the following section.

## 5. Results and Discussion

In this section, results obtained for all case studies are presented, including the different models’ configuration, RQS values, performance, and comparison with other techniques. All models were trained using Python v3.7, Tensorflow v2.1.0 and Keras v2.3.1. Hardware configuration is the following: Intel i5-9600K CPU, 16 GB DDR4 RAM and NVIDIA 11 GB Geforce RTX 2080 Ti GPU. The configurations of the trained models are shown in Table 8.

### 5.1. Case Western Reserve University Bearing Data Center (CWR)

**Figure 10:**
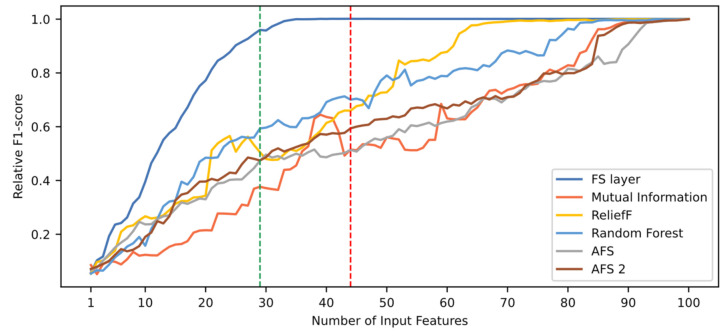
Comparison of relative F1-score based on six different rankings. The red and green dotted lines indicate the number of features needed when using the FS layer technique to reach maximum performance and a 95% of this value, respectively.

Figure 10 shows the evolution of the relative F1-score as a function of the number of features. As detailed in Section 5, features are arranged according to their importance in descending order. Regarding the use of a FS layer, it may be observed that approximately 30 features are needed for the model to reach performance values close to the maximum. After that, performance remains at a high level, reaching its maximum value with 44 variables, requiring much less variables than the rest of the techniques. When compared with other techniques, it is clearly seen that they are outperformed by the proposed technique. The difference between techniques suggests there is a large number of features with redundant information, which cannot be easily recognized by traditional methods. However, they are identified through the use of an FS layer, with their corresponding weight in the FS layer achieving a low importance value.

Table 9 shows the performance of the different techniques in terms of ranking quality and task performance. It also shows the number of variables needed to reach the maximum performance value and a 95% threshold. The RQS values presented confirm what is seen in Figure 10 regarding ranking quality. The FS layer technique achieves the highest RQS value, followed by ReliefF, Random Forest, AFS 2, Mutual Information and AFS. At some point, all techniques reach a very similar level of performance (shown in the table by $max\left(PM\right)$). However, the number of input features required for reaching the neighborhood of that value is different for each technique. The proposed technique requires 29 features to reach the 95% performance level threshold. In turn, the ReliefF, Random Forest, AFS 2, Mutual Information and AFS techniques need 63, 80, 87, 85 and 92 features, respectively. This example shows how the RQS metric favors techniques that require less variables to achieve high levels of performance. As can be seen in the figure and the table above, those techniques with the highest RQS need less input features to achieve a high level of performance. In particular, when comparing the AFS 2 technique with the Mutual Information technique, it can be seen that both reach high levels of performance needing approximately the same number of features (87 and 85, respectively). However, because the AFS 2 technique in most of the cases shows higher performance than the Mutual Information technique, it has a higher RQS.

Overall, it can be seen that in those techniques where the RQS value is higher, less features are needed to reach the 95% performance threshold. In this case, a high RQS indicates that some variables can be discarded without an important performance loss with respect to the maximum value.

Regarding performance, Table 9 shows that feature selection improves performance when using the FS layer, with a 0.14% F1-score improvement reached when using the 44 most relevant features. This suggests that the inclusion of a FS layer not only maintains the performance level, but also improves it. However, the difference in performance for this case study is not high enough as to be conclusive.

Regarding model selection, the FS layer technique is the most suitable for both criteria. In the first one, the highest performance value is reached with this technique, also with the least number of features. For the second criterion, the desired threshold is reached with only 29 features. For this criterion, the performance value is not as important as the number of features needed, because the value is above the determined threshold.

Table 10 shows the duration of the feature importance value generation process for each technique. It can be seen that the mutual information and random forest techniques achieve the best results, followed by the proposed FS layer technique. Furthermore, when the latter is compared to other embedded techniques (such as AFS and AFS 2), it is noted that the proposed technique is much less time consuming. This is due to the fact that the FS layer technique only adds one parameter per feature. This is not the case for the AFS and AFS 2 techniques, as each feature requires an attention module. As the number of features grows, the time difference between the proposed technique and the AFS techniques should also grow.

### 5.2. Commercial Modular Aero-Propulsion System Simulation (C-MAPSS): FD001

**Figure 11:**
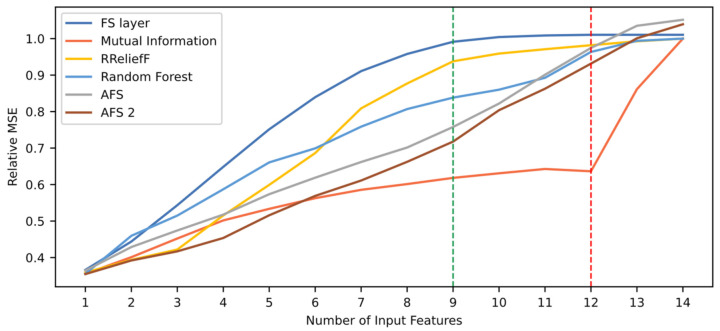
Comparison of relative MSE according to six different rankings. The red and green dotted lines indicate the number of features needed when using the FS layer technique to reach maximum performance and a 95% of this value, respectively.

Figure 11 shows the model’s relative mean squared error (MSE) according to the number of features used for testing. The proposed technique is compared to the other five techniques. As seen in the figure, the FS layer technique presents better performance than the other techniques when using from three up to 12 input features. High performance level is reached when using the top nine input features, and the maximum value is reached with 12 features. In general, the performance evolution when using the FS layer technique appears to be more stable and smoother than the other, with a linear rate at the beginning, then stabilizing at a performance value close to 1.0. On the other hand, the Mutual Information technique shows a stable behavior with low performance until testing with the top 12 features. After that, there is a sudden increase in performance. This suggests that the two least important features yielded incorrect weights by this technique. Since it does not include relationships between the input features and only the relationship of each feature with the output, it is highly possible that there are codependent features. In contrast, when looking back at the FS layer curve, the stability of the curve suggests that the technique successfully captures the dependences between input features.

To do a more complete analysis about the aforementioned issue regarding the mutual information-based technique, Table 11 shows the two most relevant features and the two least relevant features for each technique. The feature located at the middle of the ranking is also shown. Indeed, note that mutual information-based technique gives the least relevance to variables 6 (physical core speed) and 10 (corrected core speed), which are among the two most relevant features in most of the other techniques. This shows that the core speed features alone are not enough for calculating the RUL of the turbine engine. However, alongside the rest of the variables, they are highly relevant. Because of this, the mutual information-based technique is not able to recognize their importance. It is also noteworthy that the FS layer technique is the only one that gives a high importance value to feature 8 (ratio of fuel flow to HP compressor outlet), while feature 10 goes to the seventh place. This helps understand why the FS layer technique presents a performance evolution curve with a better behavior than the others.

To compare the different techniques quantitatively, Table 12 shows their ranking quality and task performance in terms of relative MSE. The results show that the proposed technique achieves higher RQS, as can be seen in Figure 11. In addition, along with the AFS and AFS 2 techniques, the proposed FS layer technique reaches a maximum performance value $\max\left(PM\right)>1$, meaning the use of this technique results in performance improvement. However, as shown in Figure 11 and Table 12, the use of the AFS and AFS 2 techniques leads to better results in terms of performance. This case serves as an example of a situation where, despite having a high RQS, a feature selection technique may lead to a maximum performance value lower than other techniques. Thus, the RQS metric does not measure performance but how fast the maximum level of performance is reached in terms of number of input features needed. Indeed, the FS layer technique presents the higher RQS value and, consequently, Table 12 shows that out of the 12 features required to achieve maximum performance, three of those can be discarded in a restricted scenario and still present a performance value within above the 95% of the maximum value. The other techniques require more features to achieve this threshold. This opens the possibility for a two-fold analysis for model selection by looking at performance and RQS simultaneously. This is useful when there is a limitation of input features to use in deployment, when the difference between performance is minimal between two models, or when performance level requirements are not so demanding. In this case study, despite the AFS techniques leading to a higher performance, the FS layer technique could be selected if there is a situation in which the number of input features is limited, as shown with the 95% threshold values. For example, when using the top nine features, the FS layer technique leads to a better performance than the rest of the techniques, and it is a value close to its maximum. In the case of the AFS techniques, the performance value with the top nine features is considerably lower than its maximum, far below the 95% threshold. This kind of information is summed up in the RQS metric, which is why it provides useful information for model selection.

Table 13 shows the time consumption for each technique when calculating the feature importance values. As with the previous case, the mutual information and random forest techniques achieve the best results, followed by the proposed technique. When comparing with the AFS techniques, the difference is not as large as with the previous case. However, it is still considerable. The AFS techniques take an order of magnitude more time than the proposed FS layer technique. This shows that despite having less variables compared to the previous case (100 against 14), the FS layer is still a faster technique.

### 5.3. Commercial Modular Aero-Propulsion System Simulation (C-MAPSS): FD004

**Figure 12:**
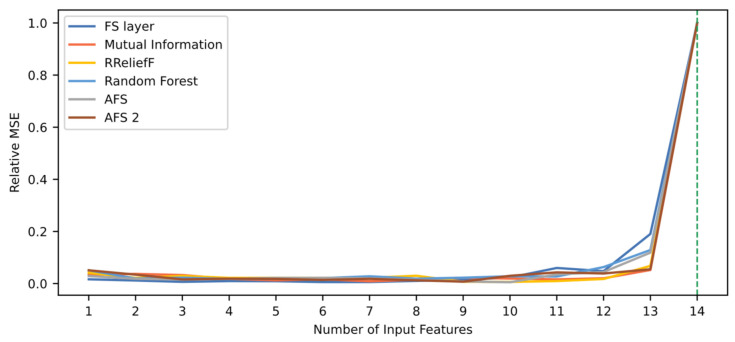
Comparison of relative MSE based on six different rankings for C-MAPSS FD004 dataset. The green dotted line indicates the number of features needed when using the FS layer technique to reach a 95% of the maximum performance value.

Figure 12 shows the relative MSE values depending on the number of variables used, based on each feature selection technique. The behavior of the curves is similar, but different to those shown in the previous two case studies since the performance does not increase unless all 14 features are used. This indicates that the individual contribution of each feature is almost zero, and none of the evaluated techniques were able to identify relations between features that lead to an improved prediction of the remaining useful life. This is opposite to the results shown using the FD001 dataset, in which performance improves as more features are added. A plausible reason is that the FD004 dataset includes six operation conditions and two failure modes, whereas the FD001 dataset only includes one of each. Therefore, the FD004 data is substantially more heterogenous. It is also possible that features have different importance values depending on the operating condition and failure mode, and that there is not a ranking capable of integrating the information of all cases within the dataset. It can be concluded that a single model for the whole dataset is not an optimal solution, and the division in subsets (and, therefore, more homogeneous data) could be a better approach. In addition, a local interpretation technique could be useful and give more insightful explanations.

Table 14 shows the performance associated to each technique, along with its ranking quality. Results show that the proposed technique achieves the higher RQS value. However, its maximum relative MSE is lower than 1. In this case, the use of an FS layer does not imply an improvement in performance. However, the difference is 0.05%; thus, it can be concluded that the same level of performance is maintained. These results are valid for the two criteria for model selection, since discarding a feature results in an important decrease in performance independently of the employed technique.

Table 15 shows how much time each technique takes to calculate the feature’s importance values. As in the two previous cases, the mutual information and random forest techniques achieve the best results, followed by the proposed technique. RReliefF, AFS and AFS 2 take longer than the rest to calculate these values. When comparing the proposed FS technique with the AFS techniques, it can be noted that the AFS techniques take more than six times longer than the proposed technique. The difference with the C-MAPSS FD001 case comes from the fact that the FD004 dataset has three times more records, and that it is trained for 10,000 epochs instead of 800.

### 5.4. Offshore Natural Gas Treatment Plant (NGTP)

**Figure 13:**
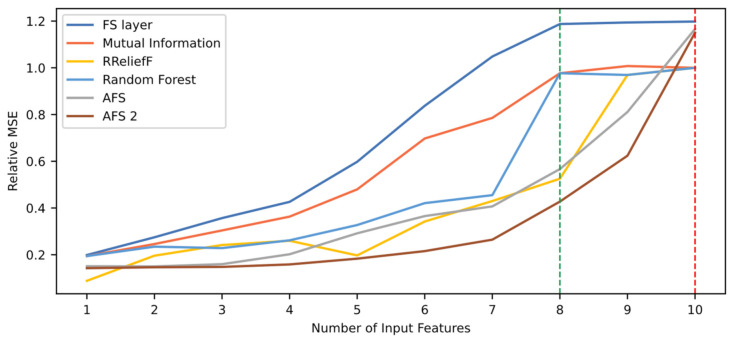
Comparison of relative MSE based on six different rankings for NGTP dataset. The red and green dotted lines indicate the number of features needed when using the FS layer technique to reach maximum performance and a 95% of this value, respectively.

Figure 13 shows how performance varies with the number features for each of the evaluated techniques. Note that the FS layer approach reaches higher performance than the others. With seven out of the 10 variables, relative MSE is >1. However, maximum performance is reached when using all 10 features.

**Figure 14:**
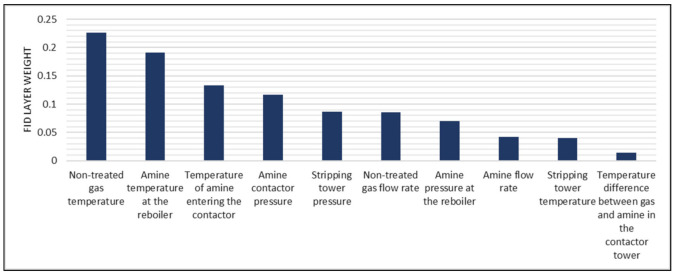
Ranking of features according to the FS layer technique.

In order to understand how the FS layer technique ranks features, Figure 14 shows the FS layer weights calculated by the model after training. It can be seen that the most relevant features are related to the amine contactor and the reboiler. Important features related to the contactor are the nontreated gas temperature, the amine temperature before entering the contactor, and the amine pressure at the contactor. This is coherent with the fact that the CO_2_ removal process occurs in the contactor and, therefore, these variables directly affect its performance. The amine temperature at the reboiler appears as the second most relevant feature, higher than stripping tower-related features. Since the heating process that occurs in the reboiler comes immediately after the stripping process, there is a correlation between them. Results in Figure 14 show that the technique is able to capture this correlation, and in the correct order. On the other hand, the least important feature is the temperature difference between nontreated gas and amine in the contactor. This means that the proposed approach can calculate internally that information from other features (i.e., nontreated gas temperature and temperature of the amine entering the contactor) and, thus, can dispose that feature. Despite the fact of the model needing all 10 features to reach its maximum relative MSE when using the FS layer technique (as seen in Figure 13), performance increments are marginal when adding the two least relevant features. A more robust neural network (with more layers or neurons per layer) could solve this issue and reach maximum performance without the need of these two last features.

Table 16 shows the quantitative comparison of the different techniques. Results show that along with the FS layer technique, the AFS techniques (AFS and AFS 2) and the Mutual Information technique led to a relative MSE higher than 1. Despite the latter reaching the maximum performance with nine variables, its value is considerably smaller than the one reached by the proposed approach. When using an FS layer, there is a performance increase of approximately 20%. Regarding ranking quality, the proposed approach has similar RQS value to the Mutual Information-based approach. This verifies what it shown in Figure 13. Although reaching different maximum values, the two curves show similar behavior with a performance improvement rate that stabilizes when using more than eight input features. On the contrary, the AFS and AFS 2 techniques reach a high maximum performance value but rely heavily on the use of all input features available. For example, when using nine out of the ten features, both techniques present the lowest performance values. Thus, despite achieving high performance, their RQS values are the lowest of all six techniques presented. This emphasizes the fact that the RQS metric does not consider the maximum performance reached but considers how fast that performance is reached in terms of input features required. This is also emphasized when looking at the results for the 95% performance threshold criterion. The AFS and AFS 2 techniques, which show the lowest RQS values, cannot discard any features from the feature set without having a major decrease in performance. This is not the case with the other techniques, in which one or two features can be discarded. In the particular case of the FS layer technique, eight features can be used for deployment with a performance decrease below 5% with respect to the maximum value and still have a better performance value than the rest of the techniques. This shows how the RQS metric helps in model selection process even when different criteria are used. The RQS indicates how likely it is to discard features without having an important performance decrease. The decision must be made by jointly analyzing performance values and RQS. After this, the corresponding criterion must be used to select the appropriate number of features.

Table 17 shows how much time each technique takes for calculating feature importance values. The same trend as in the previous cases is shown, with mutual information and random forest achieving the best results, followed by the proposed FS layer. As in the other cases, the AFS techniques take similar time to obtain these values. In this case, the AFS techniques take much more time, by a factor of more than five, for calculating feature importance values than the proposed technique. Through the comparison of the three embedded ad hoc techniques in the four case studies, it is able to see that the AFS techniques add a larger amount of complexity to the model in order to obtain feature importance values, which is reflected in the time they take.

## 6. Conclusions

In this work, a novel technique was presented for feature selection in deep neural networks for PHM models, with the objective of increasing interpretability without performance loss. It consists of a locally connected layer next to the input layer, whose weights represent the importance of the associated feature. Prior to analysis, the layer’s configuration is advantageous as it does not imply a considerable modification of the original network. In terms of trainable parameters, a number of weights equal to the number of input features is added. Regarding hyperparameters, two are added: the FS layer’s activation function and the λ term for regularization. To evaluate the quality of the ranking obtained using the FS layers weights, a new metric is introduced that analyses the feature rankings in terms of performance evolution regardless of the model’s task (it can be used for discrete health state classification as well as RUL prediction) and performance metric (it can be used for performance metrics such as accuracy, F1-score, MSE, and MAE). Thus, through the proposed framework, task performance and ranking quality are analyzed independently and simultaneously. This helps to achieve a more informed model selection.

Results across three case studies within the presented framework show that the proposed technique achieves higher RQS values than the rest of the compared techniques. It identifies irrelevant features, allowing the model to reach maximum performance with a subset of the input features. Indeed, in the CWR case, maximum performance was reached with 44 out of the 100 input features. Regarding performance, it can be concluded that the inclusion of an FS layer into a deep neural network at least maintains the same level of performance, and thus, successfully tackles the accuracy/interpretability tradeoff. Indeed, in the NGTP case, performance shows a 19.79% increase. On the other hand, results in the C-MAPSS FD004 case indicate a 0.05% decrease in RUL prediction MSE, which is inconclusive. Thus, the use of the proposed FS layer technique opens the possibility of reducing the input feature space without performance loss when using DNNs. Overall, the RQS metric proves to serve as an indicator of how likely it is to discard features without a relevant loss of performance, which is useful for real-life cases where the number of features may be a limitation and performance requirements allow a certain amount of decrease.

This work attempted to improve the interpretability of DL based PHM models and address the accuracy/interpretability tradeoff by presenting a technique for global interpretation. Even though the proposed technique allows a reduction of the feature space without performance loss and informs the importance of each feature within the model, it does not explain how features interact inside the algorithm to generate a single prediction. This is the main limitation of the proposed technique and can be addressed by enhancing it with other kinds of explanations, such as counterfactuals.

Future work includes evaluating this technique in other DL algorithms, such as autoencoders, convolutional neural networks, and long short-term memory networks to determine the effectiveness of the proposed framework in a wider range of deep learning approaches. In addition, an integration with other kinds of ML algorithms (for example, SVM) is a possible continuation of this research. To further reduce the interpretability issue mentioned in the Introduction section, it is necessary to analyze models locally. In this sense, research regarding local interpretation of DL models is also included as future work.

## Figures and Tables

**Table 1:** CWR experiment settings.

Setting	Description
Bearing Model	SKF 6205 2RSJEM
Sensors Location	Drive end bearing housing—12 o’clock position
Faults Location	Balls, outer ring, or inner ring
Motor Loads	0-1-2-3 hp
Motor Speeds	[1720,1797] rpm
Sampling Rates	48 kHz (baseline)—12 kHz (faults)

**Table 2:** CWR features’ description.

Type of Signal	Feature	Feature Number
Original signal	Maximum amplitude	1
	Root Mean Square (RMS)	2
	Peak-to-peak amplitude	3
	Crest factor	4
	Arithmetic mean	5
	Variance	6
	Skewness	7
	Kurtosis	8
	Centered moments (k = 5 − 11)	9–15
	Arithmetic mean of the Fourier amplitude, divided in 25 frequency bands	16–40
	RMS of the first five IMFs * (empirical mode decomposition)	41–45
	Percent energy of the first five IMFs (empirical mode decomposition)	46–50
	Shannon entropy of the first 5 IMFs (empirical mode decomposition)	51–55
	RMS of the first 5 PFs ** (Local Mean Decomposition)	56–60
	Percent energy of the first 5 PFs (Local Mean Decomposition)	61–65
	Shannon entropy of the first 5 PFs (Local Mean Decomposition)	66–70
Derivative of the original signal	Maximum amplitude	71
	Root Mean Square (RMS)	72
	Peak-to-peak amplitude	73
	Crest factor	74
	Arithmetic mean	75
	Variance	76
	Skewness	77
	Kurtosis	78
	Centered moments (k = 5 − 11)	79–85
Integral of the original signal	Maximum amplitude	86
	Root Mean Square (RMS)	87
	Peak-to-peak amplitude	88
	Crest factor	89
	Arithmetic mean	90
	Variance	91
	Skewness	92
	Kurtosis	93
	Centered moments (k = 5 − 11)	94–100

* IMF: intrinsic mode functions; ** PF: product functions.

**Table 3:** CWR classes’ description.

Class ID	Fault Size [mm]	Fault Location
Baseline	-	-
18BF	0.18	Balls
36BF	0.36	Balls
53BF	0.53	Balls
71BF	0.71	Balls
18IR	0.18	Inner race ring
36IR	0.36	Inner race ring
53IR	0.53	Inner race ring
71IR	0.71	Inner race ring
18OR	0.18	Outer race ring
36OR	0.36	Outer race ring
53OR	0.53	Outer race ring

**Table 4:** C-MAPSS datasets.

Dataset	Train Trajectories	Test Trajectories	Conditions	Fault Modes
FD001	100	100	1	1
FD002	260	259	6	1
FD003	100	100	1	2
FD004	249	248	6	2

**Table 5:** C-MAPSS feature descriptions.

Feature Tag	Description	Feature Tag	Description
1	Total temperature at LP * compressor outlet	8	Ratio of fuel flow to HP compressor outlet
2	Total temperature at HP ** compressor outlet	9	Corrected fan speed
3	Total temperature at LP turbine outlet	10	Corrected core speed
4	Total pressure at HP compressor outlet	11	Bypass Ratio
5	Physical fan speed	12	Bleed Enthalpy
6	Physical core speed	13	HP turbine coolant bleed
7	Static pressure at HP compressor outlet	14	LP turbine coolant bleed

* LP: Low Pressure; ** HP: High Pressure.

**Table 6:** C-MAPSS train and test sets.

Dataset	Number of Training Samples	Number of Testing Samples
FD001	20,631	13,096
FD004	61,249	41,214

**Table 7:** Monitored data for natural gas treatment plant. Source: [32].

Sensor Tag	Description	Units
A	Nontreated gas flow rate	kg/h
B	Nontreated gas temperature	°C
C	Amine contactor pressure	kPa
D	Temperature difference between nontreated gas and amine in the contactor	°C
E	Stripping tower temperature	°C
F	Stripping tower pressure	kPa
G	Amine temperature at the reboiler	°C
H	Amine pressure at the reboiler	kPa
I	Amine flow rate	kg/h
J	Temperature of amine entering the contactor	°C
K	Particles per million of CO_2_ in the treated gas	ppm

**Table 8:** Configuration of the four trained models.

Case Study	CWR	C-MAPSS		NGTP *
Dataset		FD001	FD004	
Hidden layers	2			
Neurons per hidden layer	64-32			
Activation functions	ReLU—ReLU			
Learning rate	1 × 10^−3^			
FS layer activation function	Tanh	Sigmoid	Linear	Tanh
Regularization rate	1 × 10^−6^	1 × 10^−5^	1 × 10^−3^	20,000
Epochs	2000	800	10,000	10,000

* NGTP: Natural Gas Treatment Plant.

**Table 9:** Task performance and ranking quality for CWR dataset.

Approach	RQS	$max\left(PM\right)$	Number of Variables for $max\left(PM\right)$	PM for 95% Threshold	Number of Features for 95% Threshold
FS layer	0.9754	1.0014	44	0.9599	29
Mutual Info.	0.7215	1.0000	100	0.9626	85
ReliefF	0.8630	1.0000	95	0.9587	63
Random Forest	0.8346	1.0000	98	0.9646	80
AFS	0.7127	0.9997	100	0.9629	92
AFS 2	0.7466	0.9997	100	0.9545	87

**Table 10:** Time duration for feature importance values generation (CWR case). For the embedded techniques (FS layer, AFS and AFS 2), this value is calculated as the difference between the total training time and the training time for a vanilla model without any embedded techniques. For the random forest technique, this value corresponds to the total training time, as there is no way to identify how much time it takes to obtain the desired values.

Approach	FS Layer	Mutual Info.	ReliefF	Random Forest	AFS	AFS 2
**Time [s]**	89.3	5.2	595.3	10.2	2377.0	3026.5

**Table 11:** Features ranked 1st, 2nd, 7th, 13th and 14th in the ranking, for the six compared techniques.

Ranking	FS Layer	Mutual Info.	RReliefF	R. Forest	AFS	AFS 2
1st	8	7	**10**	7	**10**	**10**
2nd	**6**	3	**6**	**6**	7	**6**
7th	**10**	13	8	11	4	12
13th	5	**6**	12	5	12	2
14th	2	**10**	14	12	8	11

**Table 12:** Task performance and ranking quality for C-MAPSS FD001 dataset.

Approach	RQS	$max\left(PM\right)$	Number of Features for $max\left(PM\right)$	PM for 95% Threshold	Number of Features for 95% Threshold
FS layer	0.9216	1.0104	12	0.9913	9
Mutual Info.	0.6786	1.0000	14	1.0000	14
RReliefF	0.8730	1.0000	14	0.9589	10
Random Forest	0.8465	1.0000	14	0.9634	12
AFS	0.7821	1.0515	14	1.0351	13
AFS 2	0.7560	1.0392	14	1.0007	13

**Table 13:** Time duration for feature importance values generation (C-MAPSS FD001 case).

Approach	FS Layer	Mutual Info.	RReliefF	Random Forest	AFS	AFS 2
**Time [s]**	53.9	7.3	503.3	26.7	316.9	322.9

**Table 14:** Task performance and ranking quality for C-MAPSS FD004 dataset.

Approach	RQS	$max\left(PM\right)$	Number of Features for $max\left(PM\right)$	PM for 95% Threshold	Number of Features for 95% Threshold
FS layer	0.1750	0.9995	14	0.9995	14
Mutual Info.	0.1524	1.0000	14	1.0000	14
RReliefF	0.1545	1.0000	14	1.0000	14
Random Forest	0.1715	1.0000	14	1.0000	14
AFS	0.1679	0.9761	14	0.9761	14
AFS 2	0.1726	0.9652	14	0.9652	14

**Table 15:** Time duration for feature importance values generation (C-MAPSS FD004 case).

Approach	FS Layer	Mutual Info.	RReliefF	Random Forest	AFS	AFS 2
**Time [s]**	1435.1	120.0	6347.0	120.7	9083.7	9124.3

**Table 16:** Task performance and ranking quality for the NGTP case study.

Approach	RQS	$max\left(PM\right)$	Number of Features for $max\left(PM\right)$	PM for 95% Threshold	Number of Features for 95% Threshold
FS layer	0.7755	1.1979	10	1.1873	8
Mutual Info.	0.7582	1.0075	9	0.9767	8
RReliefF	0.5676	1.0000	10	0.9693	9
Random Forest	0.6596	1.0000	10	0.9767	8
AFS	0.4946	1.1664	10	1.1664	10
AFS 2	0.4126	1.1503	10	1.1503	10

**Table 17:** Time duration for feature importance values generation (NGTP case).

Approach	FS Layer	Mutual Info.	RReliefF	Random Forest	AFS	AFS 2
**Time [s]**	188.2	0.5	237.8	5.4	1059.6	1071.0

## Data Availability

CWR dataset is available in [72]. C-MAPSS datasets are available in [88].

## Appendix A

To prove that

$$\frac{\sum_{n=1}^{N}PM_{n}\cdot n}{\sum_{n=1}^{N}\max\left(PM\right)\cdot n}\leq 1\;\Leftrightarrow \;\overset{N}{\underset{n=1}{\sum }}PM_{n}\cdot n\leq \overset{N}{\underset{n=1}{\sum }}\max\left(PM\right)\cdot n$$

mathematical induction was used. We generalized $PM_{n}$ to any function $\alpha \left(n\right)$ with values within the range [0,1].

**Base case (n=1).**

$$\overset{1}{\underset{n=1}{\sum }}\alpha \left(n\right)\cdot n=\alpha \left(1\right)\cdot 1\leq \underset{n\in \left[1,1\right]}{\max}\left\{\alpha \left(n\right)\right\}\cdot 1=\overset{1}{\underset{n=1}{\sum }}\underset{n\in \left[1,1\right]}{\max}\left\{\alpha \left(n\right)\right\}\cdot n$$

**Induction step.**

$$\overset{K}{\underset{n=1}{\sum }}\alpha \left(n\right)\cdot n\leq \overset{K}{\underset{n=1}{\sum }}\underset{n\in \left[1,K\right]}{\max}\left\{\alpha \left(n\right)\right\}\cdot n\Rightarrow \overset{K+1}{\underset{n=1}{\sum }}\alpha \left(n\right)\cdot n\leq \overset{K+1}{\underset{n=1}{\sum }}\underset{n\in \left[1,K+1\right]}{\max}\left\{\alpha \left(n\right)\right\}\cdot n$$

**Proof.** 

$$\overset{K+1}{\underset{n=1}{\sum }}\alpha \left(n\right)\cdot n=\overset{K}{\underset{n=1}{\sum }}\alpha \left(n\right)\cdot n+\alpha \left(K+1\right)\cdot \left(K+1\right)\;\leq \overset{K}{\underset{n=1}{\sum }}\underset{n\in \left[1,K\right]}{\max}\left\{\alpha \left(n\right)\right\}\cdot n+\alpha \left(K+1\right)\cdot \left(K+1\right)\;=\underset{n\in \left[1,K\right]}{\max}\left\{\alpha \left(n\right)\right\}\cdot \frac{K\left(K+1\right)}{2}+\alpha \left(K+1\right)\cdot \left(K+1\right)\;\leq \underset{n\in \left[1,K+1\right]}{\max}\left\{\alpha \left(n\right)\right\}\cdot \frac{K\left(K+1\right)}{2}+\underset{n\in \left[1,K+1\right]}{\max}\left\{\alpha \left(n\right)\right\}\cdot \left(K+1\right)\;=\underset{n\in \left[1,K+1\right]}{\max}\left\{\alpha \left(n\right)\right\}\left(\frac{K\left(K+1\right)}{2}+\left(K+1\right)\right)\;=\underset{n\in \left[1,K+1\right]}{\max}\left\{\alpha \left(n\right)\right\}\left(\frac{\left(K+1\right)\left(K+2\right)}{2}\right)\;=\overset{K+1}{\underset{n=1}{\sum }}\underset{n\in \left[1,K+1\right]}{\max}\left\{\alpha \left(n\right)\right\}\cdot n$$

 □
